# Commensal gut bacteria modulate phosphorylation-dependent PPARγ transcriptional activity in human intestinal epithelial cells

**DOI:** 10.1038/srep43199

**Published:** 2017-03-07

**Authors:** Malgorzata Nepelska, Tomas de Wouters, Elsa Jacouton, Fabienne Béguet-Crespel, Nicolas Lapaque, Joël Doré, Velmurugesan Arulampalam, Hervé M. Blottière

**Affiliations:** 1 Micalis Institute, INRA, AgroParisTech, Université Paris-Saclay, 78350 Jouy-en-Josas, France; 2 Laboratory of Food Biotechnology, Institute of Food, Nutrition and Health, ETH Zurich, Switzerland; 3 MGP MetaGenoPolis, INRA, Université Paris-Saclay, 78350 Jouy en Josas, France; 4 Karolinska Institutet, Department of Microbiology, Tumor and Cell Biology (MTC), Stockholm, Sweden

## Abstract

In healthy subjects, the intestinal microbiota interacts with the host’s epithelium, regulating gene expression to the benefit of both, host and microbiota. The underlying mechanisms remain poorly understood, however. Although many gut bacteria are not yet cultured, constantly growing culture collections have been established. We selected 57 representative commensal bacterial strains to study bacteria-host interactions, focusing on PPARγ, a key nuclear receptor in colonocytes linking metabolism and inflammation to the microbiota. Conditioned media (CM) were harvested from anaerobic cultures and assessed for their ability to modulate PPARγ using a reporter cell line. Activation of PPARγ transcriptional activity was linked to the presence of butyrate and propionate, two of the main metabolites of intestinal bacteria. Interestingly, some stimulatory CMs were devoid of these metabolites. A *Prevotella* and an *Atopobium* strain were chosen for further study, and shown to up-regulate two PPARγ-target genes, ANGPTL4 and ADRP. The molecular mechanisms of these activations involved the phosphorylation of PPARγ through ERK1/2. The responsible metabolites were shown to be heat sensitive but markedly diverged in size, emphasizing the diversity of bioactive compounds found in the intestine. Here we describe different mechanisms by which single intestinal bacteria can directly impact their host’s health through transcriptional regulation.

Much effort has been put into studying the interactions between humans and microbes, focusing on mechanism of pathogenicity in infectious diseases. Current knowledge on subtle interactions between commensal bacteria and their host is scarce, in spite of the increased awareness of their importance for wellbeing and in the onset of chronic diseases^1^. Finely tuned interactions between the gut microbiota and the host’s intestinal tissues are widely considered to be responsible for the establishment of an equilibrium state, ranging from commensalism to mutualism^2^. The gut microbiota is at a key interface between food and the host. With its large genetic pool it contributes to a multitude of intestinal functions, ranging from digestion of complex polysaccharides, production of essential nutrients or vitamins, and regulation of host fat storage, to reinforcing the barrier function against pathogens, and the maturation of the immune system^3^,^4^,^5^,^6^,^7^. Strong links between the gut microbiota, low-grade inflammation and host metabolism have been highlighted recently^8^,^9^. However, the underlying mechanisms by which the gut microbiota can contribute to the host’s metabolic and immune homeostasis or dysfunction remain elusive. An important role has been attributed to short chain fatty acids produced in the colon by bacterial fermentation of dietary fibres, linking immune system and energy intake, but several other known metabolites may also play a role^10^, as well as many others yet to be identified^11^,^12^.

PPARγ is a nuclear receptor for which lipids and their metabolic products are known ligands^13^. Two isoforms of PPARγ are known, PPARγ1 and PPARγ2. They both form heterodimers with the retinoid X receptor (RXR) that regulate transcription through binding to PPAR-responsive elements (PPREs) in target-gene promoters. PPARγ is strongly expressed in the colon^14^ where it has been shown to be highly involved in the colonocyte’s metabolic regulation, cell cycle, cell differentiation and inflammation^15^,^16^,^17^. At a systemic level, intestinal PPARγ has been shown to impact various patho-physiological conditions linked to the intestinal microbiota. It affects lipid storage in adipose tissues through transcriptional regulation of ANGPTL4 in the intestine^18^. Moreover, accumulating evidence links PPARγ to chronic inflammatory conditions and diabetes. Treatment with specific PPARγ agonists reduced intestinal inflammation^19^ as well as colon cancer development^16^,^20^,^21^ and type 2 diabetes^22^.

In the present study we aimed to identify commensal gut bacteria able to regulate this crucial nuclear receptor using commensal bacterial cultures and PPARγ-dependent reporter cells. Moreover, we attempted to decipher the underlying molecular mechanism by which commensals can impact this pathway in intestinal epithelial cells (IECs) and characterized the activating compounds size and heat stability.

## Results

### Bacterial metabolites modulate PPARγ-dependent transcriptional activity in HT-29 cells

**Figure 1:**
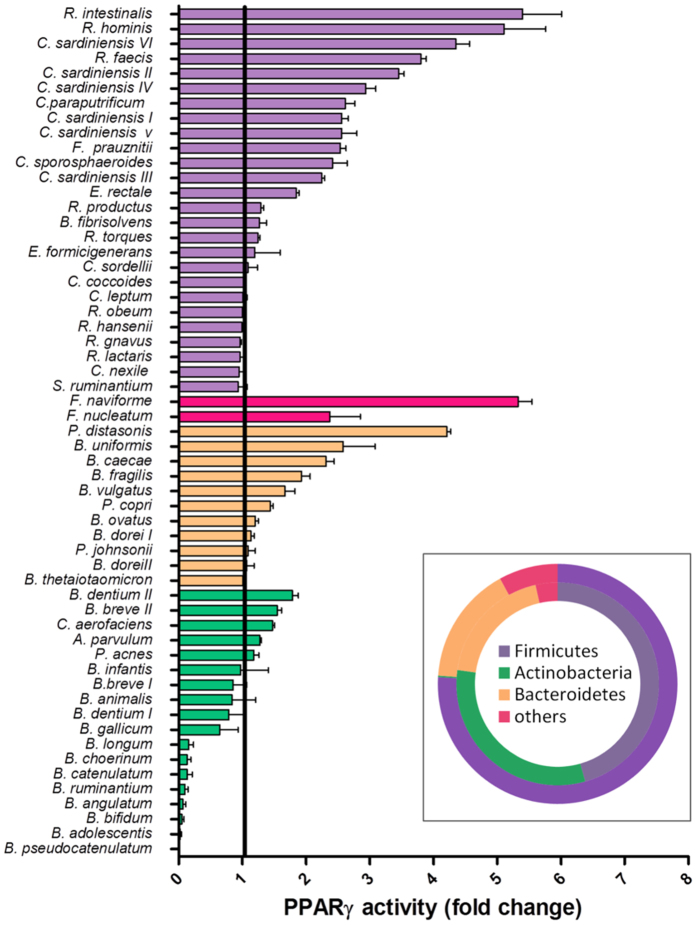
Effect of conditioned media (CM) on transcriptional activity of PPARγ in HT-29-PPARγ reporter cells. Activation expressed as the fold increase towards its control (growth-medium) are represented as bar plot. Bacteria are sorted by response in decreasing order and grouped by phylum (violet = Firmicutes, pink = Fusobacteria, yellow = Bacteroidetes, green = Actinobacteria). Distribution of the bacterial phyla is represented in the doughnut chart on the bottom right. The inner circle represents the screened collection, the outer circle the phyla representation in the human colon based on the MetaHIT data.

Fifty-seven gut bacterial strains belonging to the major phyla in the human intestine, Firmicutes, Bacteroides, Actinobacteria and Fusobacteria (Fig. 1), were screened for their potential to modulate PPARγ activity in human IECs. Due to the thick mucus layer, bacteria-host interactions in the colon are thought to be largely mediated by secreted compounds. Therefore we chose to test conditioned media (CMs) of the selected strains, *i.e.* filtered bacterial culture media after growth to stationary phase^23^,^24^. All bacteria were cultured anaerobically to favour the expression of genes likely to play a role in their anaerobic habitat, the human gut. An HT-29-PPARγ reporter cell line was used to identify bioactive bacterial products involved in PPARγ regulation in the gut epithelium. HT-29 is a well-characterized epithelial cell line with colonocytic differentiation characteristics. CMs showed species-specific PPARγ activation capacity (Fig. 1). Although reporter gene activities were not strictly correlated to phylogenetic affiliation of the strains, the strongest overall stimulatory effect was observed among Firmicutes and Fusobacteria, while Actinobacteria exerted moderate or no modulation (Supplementary Fig. 1). Some Actinobacteria caused cell detachment and thus lower luciferase activity, due to acidification of the culture medium by CM.

*Roseburia hominis, Roseburia intestinalis* and *Fusobacterium naviforme* displayed the strongest activation-potential, causing a 5-fold increase (5.11 ± 1.4, 5.4 ± 1.3, and 5.3 ± 0.4, respectively) of PPARγ reporter activity (Supplementary Table 1). As *Roseburia* and *Fusobacterium* are well-documented producers of butyrate (the concentrations of butyrate in conditioned media were among the highest measured with 8.9, 11.7 and 23.9 mM respectively), we hypothesized that the response pattern of our PPARγ reporter cells might be related to the organic acids composition of our CMs.

### Butyrate is a major driver of PPARγ-dependent transcriptional response in IECs

It is well known that SCFA, especially butyrate, play an important role in gene regulation in intestinal epithelial cells^25^,^26^,^27^. We quantified the concentrations of different organic acids (OA, that is formate, acetate, propionate, butyrate, succinate and lactate) in the CMs using HPLC and GC-MS (Supplementary Table 1). Correlation of these single OA in CMs with their PPARγ-activation capacity indicates a potential role of butyrate (spearman correlation factor 0.69) and propionate (spearman correlation factor 0.41) (Supplementary Fig. 2). These two metabolites occur independently since butyrate and propionate rarely co-occur in our CMs (spearman correlation factor 0.02). Acetate had a negative effect on the PPARγ reporter system displaying an inverse correlation (spearman correlation factor −0.35).

**Figure 2:**
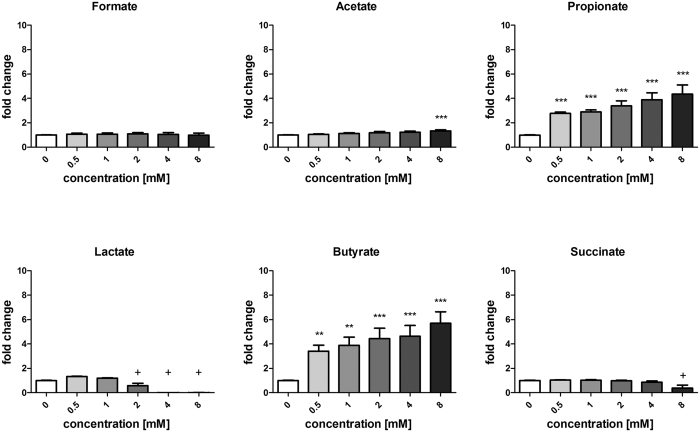
Dose-response of organic acids and SCFA on PPARγ activation. Fold change represents the readout of the reporter cell line (relative light units: RLU) normalized to the non-treated control. HT-29-PPARγ cells were exposed to formate, succinate, lactate, acetate, propionate, butyrate in concentrations rising from 0.5 to 8 mM for 24 h. Data are represented as mean ± standard error of the mean (SEM) of triplicate measurement of three independent experiments. ***P < 0.001, **P < 0.005, compared with the control (Student’s t-test). ^+^Significant decrease due to impaired cell viability.

We confirmed the modulatory potential of the different OA applying 1/10 dilutions of the CMs to the PPARγ reporter cell line (Fig. 2). The concentrations correspond to the non-cytotoxic dilutions of fecal waters^28^,^29^ and can thus be considered as physiological concentrations. Consistently with the correlation analysis, only butyrate and propionate strongly stimulated PPARγ activity in a dose-dependent manner. A significant activation by butyrate or propionate was observed at concentrations as low as 0.5 mM (which corresponds to a tested CM containing 5 mM), resulting in a 2.25 ± 0.1 and 2.93 ± 0.4 fold stimulation for CMs containing 3.75 and 5.98 mM butyrate, respectively, but no propionate. A slight activation was observed with 8 mM acetate, the highest concentration tested (Fig. 2). However the highest measured acetate concentration in the CMs was below the activating concentration at 61 mM in the *B. pseudocatenulatum* CM (Supplementary Table 1). At low doses, lactate had no effect but showed cytotoxicity starting at 2 mM resulting in cell detachment. The other acids had no effect on our PPARγ reporter system except a toxic effect at high concentrations of succinate (8 mM, Fig. 2). Overall, low doses of butyrate and propionate stimulate PPARγ activation while high concentrations of all OA especially acetic and lactic acid have a detrimental effect on cell viability probably due to the associated pH decrease.

### PPARγ-dependent gene activation clusters with organic acid (OA) profiles

**Figure 3:**
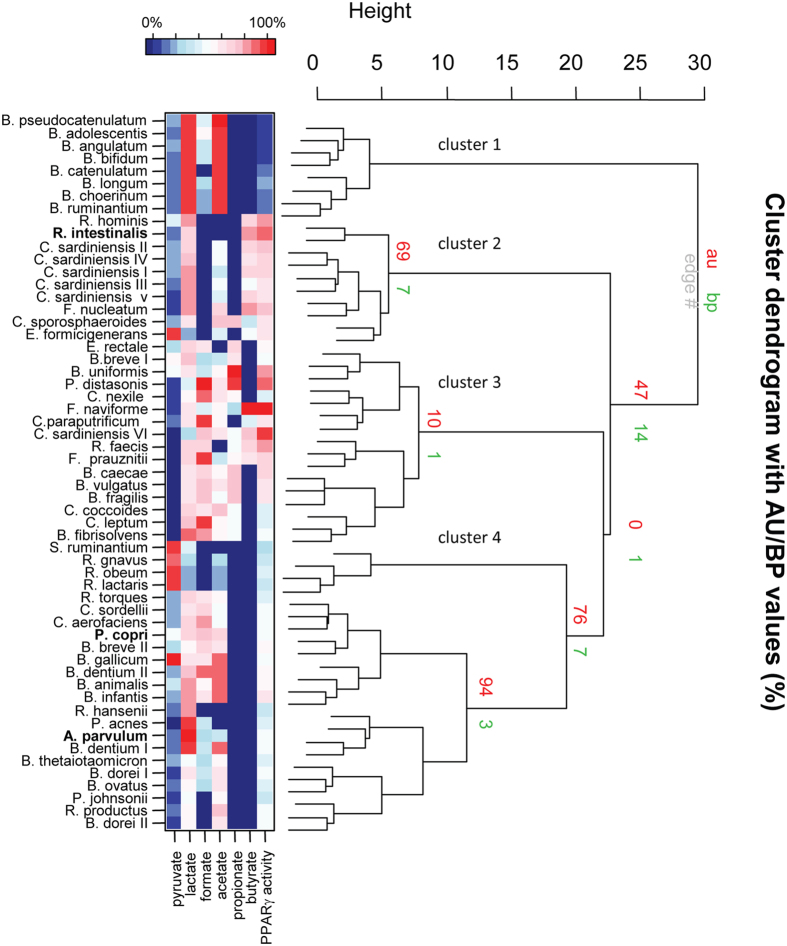
Bacterial species and their OAs pattern cluster with PPARγ response. Clustering using pvclust with AU (Approximately Unbiased) p-value and BP (Bootstrap Probability). The heat-map represents the PPARγ response and OA concentrations in percent of the highest value for the respective variable.

**Figure 4:**
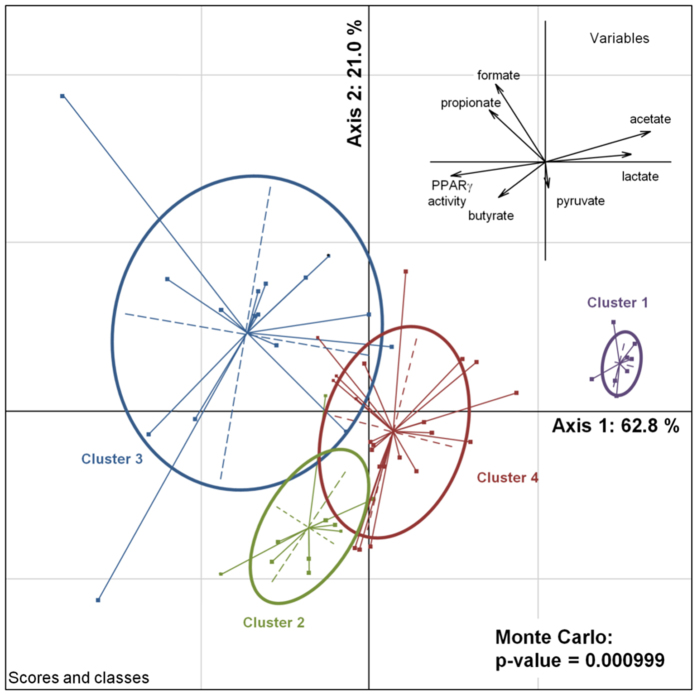
Inter-class PCA using classes defined by previous cluster analysis. The PCA separates the 4 clusters significantly (Monte-Carlo test: p = 0.000999). Variables are plotted on the same components (upper right of the graph) showing their importance in the separation of the clusters.

The rather weak but clear correlations between the single OAs and PPARγ-dependent luciferase activity (Supplementary Fig. 2) led us to hypothesize that the action of these OAs might depend on their combination rather than on a single OA. For instance, high butyrate production resulted in low activation if combined with high concentrations of lactate, as observed in the CMs of *F. nucleatum* and *C. sardiniensis*. To identify combinations of OAs influencing PPAR*γ*-activation, we performed cluster analysis of the screening results using OA concentrations and PPAR*γ*-activation as parameters (Fig. 3). Clustering revealed 4 main clusters based on the selected parameters (Fig. 3). In order to identify the driving forces for the separation of these clusters we performed an inter-class PCA (Fig. 4). It confirmed a significant separation of the 4 clusters (p-value = 0.000999, Monte-Carlo significance test using 1000 replicates) and indicated the main driving forces for cluster separation. Cluster 1 is driven by high concentrations of lactate and acetate known to be toxic for the HT-29 cells and therefore inversely correlating with PPARγ activity. Clusters 2 and 3 are the main activating clusters. While cluster 2 is dominated by the presence of butyrate, cluster 3 shows the presence of propionate, absent in cluster 2. Low activating cluster 4 is characterized by the absence of propionate and butyrate and, in opposition to cluster 1, by moderate levels of lactate and acetate. Interestingly, we can observe single CMs deprived of propionate and butyrate with low-level activation in cluster 4 (see activation per cluster in Supplementary Fig. 3).

### Commensal bacteria activate PPARγ-dependent transcription in different ways

**Figure 5:**
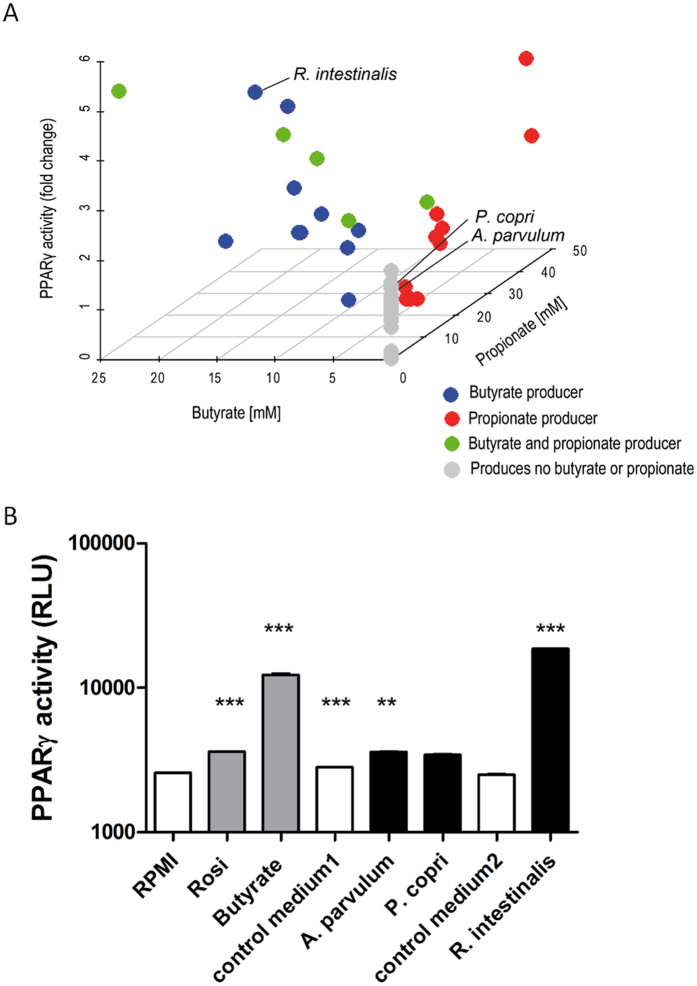
SCFA independent activators of PPARγ (**A**) PPARγ activation plotted against butyrate and propionate concentrations. CMs containing butyrate but no propionate are represented in blue, CMs containing propionate but no butyrate are represented in red, CMs containing both propionate and butyrate are represented in green and CMs deprived of these two SCFA are represented in grey. (**B**) Activation of PPARγ pathway by chosen bacteria on colonic reporter cell line HT-29-PPARγ. Rosiglitazone (Rosi, 5 μM) is used as control for activation. Control medium 1 is the medium used to culture of *A. parvulum* and *P. copri* (M104). Control medium 2 is the medium used to culture *R. intestinalis* (M58). Data are represented as mean ± standard error of the mean (SEM) of triplicate measurement of a representative of three independent experiments. ***P < 0.001, **P < 0.005, compared with the control media (Student’s t-test).

Inter-class analysis showed two SCFA, butyrate and propionate, as main driving forces of PPAR*γ* activation in the tested CMs. Plotting the activation of all CMs relative to their propionate and butyrate content, we visualized a group of low activating CMs showing bioactivity independently of butyrate or propionate (Fig. 5A). We chose to further investigate this group using *Atopobium parvulum* and *Prevotella copri* as activating strains without butyrate or propionate in their CMs. *Roseburia intestinalis* was chosen as butyrate-producing control strain for further studies. We first confirmed the screening results in three independent new cultures (Fig. 5B), showing that the activation by *A. parvulum* and *P. copri* was in the range of a specific activation of PPARγ by the ligand rosiglitazone (10 μM). The butyrate-producing strain *R. intestinalis* in contrast, showed a much higher activation comparable to that of butyrate (2 mM).

### *A. parvulum* and *P. copri* induce the expression of PPARγ target genes in HT-29 cells

**Figure 6:**
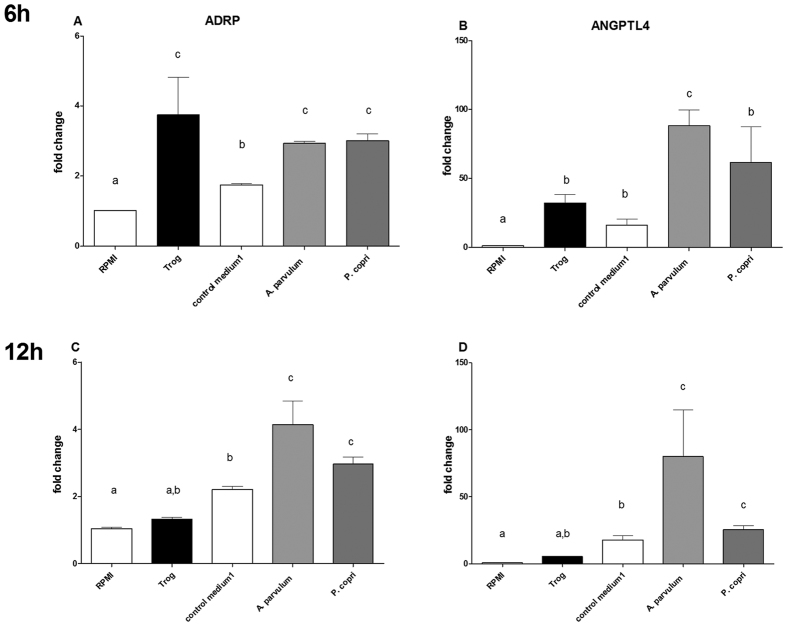
Transcriptional regulation of PPARγ target genes upon stimulation with chosen CMs. Up-regulation of mRNA for ADRP (**A,C**) and ANGPTL4 (**B,D**) by chosen CMs. The expression determined by Quantitative real-time PCR on total RNA extracted from cells exposed to CMs for 6 h (**A,B**) and 12 h (**C,D**) higher and lower panel respectively. Expression is represented as fold change compared to the absence of any stimulation (RPMI cell culture medium only). Data are represented as mean ± standard error of the mean (SEM) of triplicates of one representative experiment of three independent repetitions. Data were analyzed applying an ANOVA test followed by a post-hoc Tuckey test. Bars superscripted with different letters have a difference of at least p < 0.05.

To assess the impact of PPARγ activation by chosen bacterial CMs, we quantified the expression of two well-known PPARγ target genes: adipose differentiation-related protein (*ADRP*) and angiopoietin-like protein 4 (*ANGPTL4*), using RT-qPCR. Although the latter has previously been shown to be activated by butyrate through a PPARγ independent mechanism^25^, its regulation through PPARγ by the intestinal microbiota has an important physiological impact^30^. Time points of gene expression evaluation were set to 6 and 12 h in order to account for both direct and indirect activation of PPARγ. The stimulation of HT-29 cells with CMs of *A. parvulum* resulted in an 88.0 ± 8 fold increase of ANGPTL4 expression after 6 h (Fig. 6B). Stimulation with CMs of *P. copri* reached statistical significance only after 12 h (Fig. 6D), suggesting a different mechanism of action than the one exhibited by *A. parvulum.* In both cases, the induction of *ANGPTL4* was stronger than that observed with the PPARγ-specific ligand troglitazone. After 12 h, the levels of ANGPTL4 and ADRP expression were still higher than after stimulation by troglitazone.

### *A. parvulum* and *P. copri* induce PPARγ phosphorylation through ERK1/2

**Figure 7:**
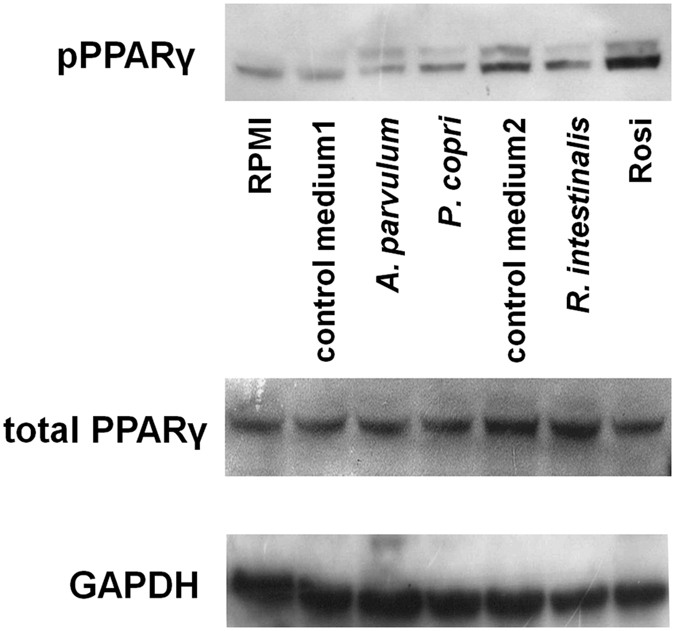
Phosphorylation of PPARγ by *A. parvulum* and *P. copri* supernatants. *A. parvulum, P. copri* and rosiglitazone (10 μM) induce PPARγ phosphorylation as compared to its growth medium M104. The medium M58 used for the culture of *R. intestinalis* shows strong activation capacity itself and therefore no increase of PPARγ phosphorylation can be observed. The nuclear fraction proteins were blotted (Western Blot) for phosphorylated PPARγ. Total PPARγ was used as control for phosphorylated PPARγ. GAPDH was used as loading control.

**Figure 8:**
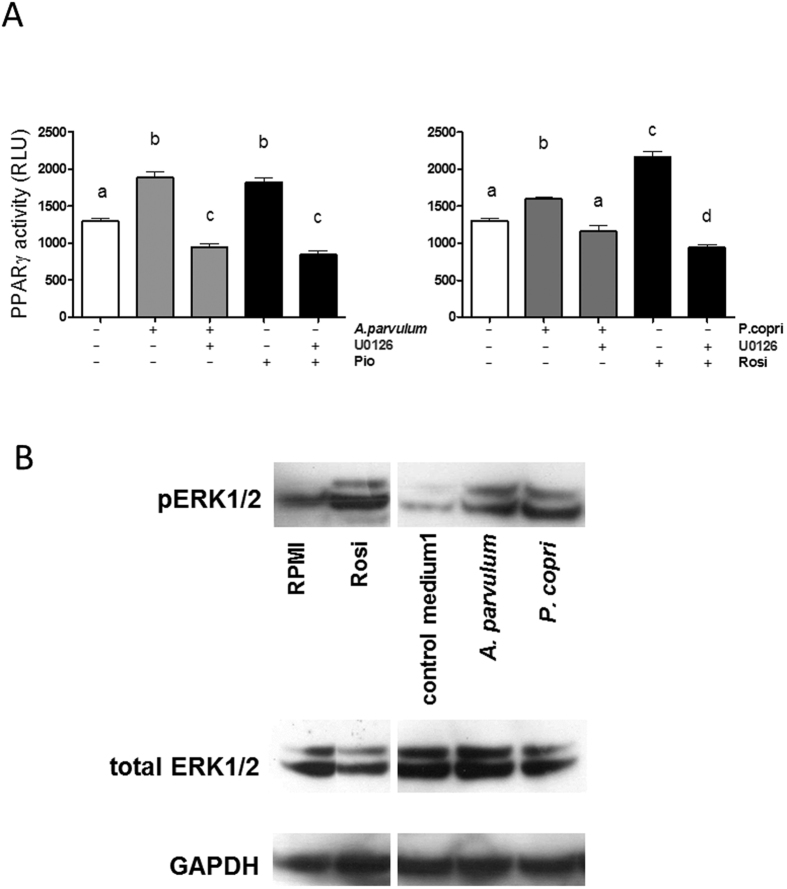
Implication of ERK in the activation of PPARγ. (**A**) The *A. parvulum* and *P. copri* -induced signal were inhibited using the specific inhibitor for MEK/ERK (U0126) on HT-29-PPARγ cells. Different letters indicate statistically different results (p < 0.05). (**B**) The implication of MEK/ERK in PPARγ activation by *A. parvulum* and *P. copri* was confirmed on the protein level showing phosphorylation of ERK1/2. Total ERK1/2 was used as control for phosphorylated PPARγ. GAPDH was used as loading control.

Phosphorylation of PPARγ has previously been proposed as a mechanism for bacterial activation of PPARγ in intestinal epithelial cells^31^. We therefore assessed the effect of *A. parvulum* and *P. copri* on PPARγ phosphorylation in HT-29 cells. After 2 h, HT-29 cells exposed to CMs of either *A. parvulum* or *P. copri* showed an increase in phosphorylated PPARγ as compared to cells exposed to the bacterial culture medium M104 (DSMZ Medium 104, further referred to as medium 1) (Fig. 7). HT-29 cells exposed to the CM of *R. intestinalis* in contrast, showed a lower phosphorylation status of PPARγ than cells exposed to the bacterial culture medium (DSMZ Medium 58, further referred to as medium 2), which itself caused a high induction of PPARγ phosphorylation but no luciferase gene activation (Fig. 5B). This result suggests that the butyrate-dependent effect of *R. intestinalis* may not depend on PPARγ phosphorylation, but more likely depends on a butyrate driven HDAC inhibitory effect, leading to the increased expression of a variety of genes in intestinal epithelial cells^25^. The activation of PPARγ by *A. parvulum* and *P. copri* through phosphorylation of PPARγ was further studied in order to identify the cause for this phosphorylation. We assessed the role of ERK1/2, which is known to mediate PPARγ phosphorylation^32^ using a specific ERK1/2 inhibitor (U0126). U0126 abolished the activation of PPARγ by CMs of both *A. parvulum* and *P. copri*, as well as by the PPARγ agonists rosiglitazone and pioglitazone (Fig. 8A), implicating ERK1/2 in the observed effect. To further confirm the involvement of MEK/ERK in the activation of PPARγ, we assayed whole cell extracts for ERK1/2 kinase by Western Blotting (Fig. 8B). After 30 min of activation with the studied bacterial CMs, ERK1/2 was highly phosphorylated, similar to what was observed with rosiglitazone.

### *A. parvulum* and *P. copri* induce PPARγ activation by differential molecules

**Figure 9:**
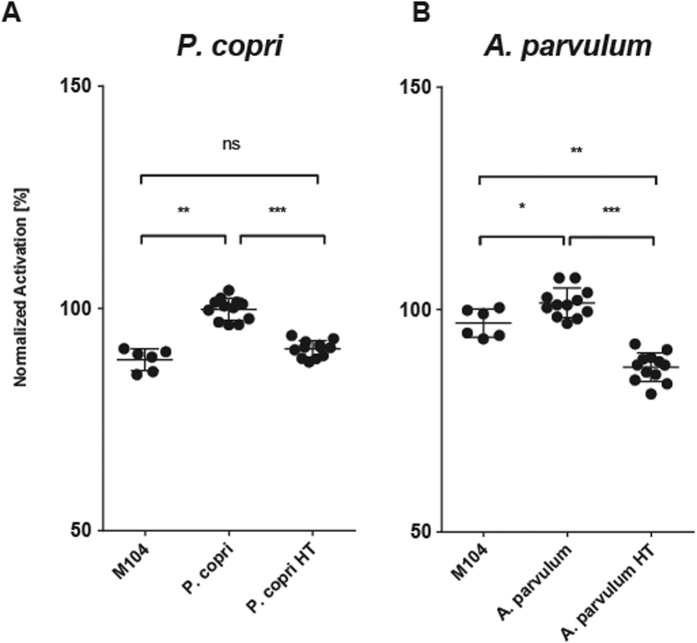
Loss of PPARγ activation upon heat treatment of conditioned media. *P. copri* (**A**) and *A. parvulum* (**B**) conditioned media were incubated for 10 min at 100 °C to test the heat-stability of the PPARγ activating compound. In comparison with the culture medium M104 both *P. copri* (**A)** and *A. parvulum* (**B**) show significant activation of CM. The activation of PPARγ is lost after the heat treatment (HT). Experiments were performed in triplicates using two independent bacterial cultures and normalized to the activating CMs indicated as 100%. Data were analyzed applying an ANOVA test followed by a post-hoc Tuckey test. Significance levels are indicated as follows: ***P < 0.001, **P < 0.005, *P < 0.05.

**Figure 10:**
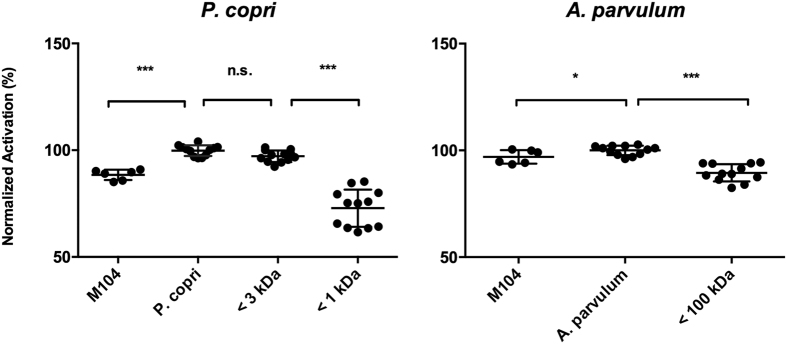
Identification of the size of PPARγ activating compound in the CMs. *A. parvulum* and *P. copri* CMs were fractions into fractions >100 kDa, >50 kDa, >30 kDa, >10 kDa, >3 kDa and >1 kDa. The lowest fraction showing activation on PPARγ reporter cell lines and the highest fraction showing loss of activity are represented for *P. copri* (**A**) and *A. parvulum* (**B**). The activating compound produced by *P. copri* is smaller than 3 kDa and bigger then 1 kDa. *A. parvulum* loses activity significantly after filtering using a 100 kDa filter. Experiments were performed in triplicates using two independent bacterial cultures and normalized to the activating CMs. Data were analyzed applying an ANOVA test followed by a post-hoc Tuckey test. Significance levels are indicated as follows: ***P < 0.001, **P < 0.005, *P < 0.05.

In order to better characterize the nature of the PPARγ activating compounds produced by *A. parvulum* and *P. copri* we performed heat stability and a size determination tests. Both CMs lost a significant part of their PPARγ activation capacity after heat treatment (p < 0.001 for both). The heat-treated *P. copri* CM lost its effect, reducing activation to 90 ± 1.8% of that observed with the untreated sample, a level comparable to that observed with the control medium M104 which activates to 88.5 ± 2.2% of the level observed with *P. copri* CM. Activation by *A. parvulum* was reduced to 87 ± 3% of the activation by the untreated CM, below the baseline activation by the control medium M104 (96.9 ± 2.9%) (Fig. 9). Hence the active compound is in both cases sensitive to heat treatment, suggesting a molecule that is denaturated or hydrolysed, or evaporates. The bioactive compounds of *P. copri* and *A. parvulum* can clearly be differentiated by their size. While the active compound of *P. copri* was present in a seize-filtered fraction between 1 and 3 kDa, the activating fraction of *A. parvulum* was estimated above 100 kDa (Fig. 10), indicating that both activating compounds are different molecules and thus suggesting different activation mechanisms.

## Discussion

The gut and its colonizing microbiota provide the host with many important biological functions essential for human physiology^33^. This demands a balanced interaction between the host and its microbiota, mediated through direct contact as well as through secreted bioactive compounds. The present study supports the growing awareness of a direct impact of the microbiota on host physiology, ultimately maintaining gut homeostasis^34^. The ability of the microbiota to affect such a versatile nuclear receptor as PPARγ further emphasizes the strong interconnection of metabolic and immunomodulatory regulation in the human intestine.

We used a colonic epithelial cell line engineered to monitor the transcription modulation activity of PPARγ in response to bacterial metabolites. Upon preparation of CMs of 57 commensal bacteria grown under anaerobic conditions, the major bacterial metabolites (OAs) as well as pH and OD600 were quantified. The results of the cellular assay revealed a correlation between butyrate or propionate contents in the CMs and PPARγ-dependent transcriptional activity in accordance with previous reports^27^. The dose-dependent activation potential of butyrate and propionate on the PPARγ reporter was confirmed (Fig. 2). The results of screening the 57 CMs in the PPARγ reporter assay subsequently stratified modulating bacteria into: (i) activators through known activating SCFAs (butyrate and propionate), (ii) non-activators or inhibitors and (iii) a group showing activation through mechanisms that were not clearly linked to either butyrate or propionate.

Since the important role of SCFAs in epithelial health, proliferation and differentiation has repeatedly been confirmed, we used cluster analysis to test if the observed third group might be a result of combinations of bacterial OA (Fig. 3). An inter-class analysis of the obtained clusters confirmed that the cluster 4 - which does not contain any OAs tested positive for PPARγ activation - indeed is driven by none of the quantified parameters (Fig. 4). We identified four significantly different clusters driven either by their activation potential through butyrate or propionate (clusters 2 and 3) or by their inhibitory effect through high acetate and lactate concentrations, and finally cluster 4 which groups neutral and activating supernatants lacking activation driving components within the quantified parameters. This clear separation encouraged additional investigation of the molecular pathways involved in PPARγ activation by commensal bacteria of the last and previously undescribed group. We confirmed the SCFA independent activation for two bacteria of this group: *A. parvulum* and *P. copri* that were subsequently further studied. *R. intestinalis* was chosen as a control for activation through butyrate (Fig. 5). The PPARγ activation by *A. parvulum* and *P. copri* in the reporter cell lines were confirmed by the transcriptional up-regulation of ADRP and ANGPTL4 in parental HT-29 cells (Fig. 6). Both genes are well known targets of PPARγ in the intestinal epithelium, with a systemic impact on metabolism^30^. The observed up-regulation of PPARγ activity has previously been described for strains of *Enterococcus faecalis* isolated from new born babies^31^. In that study, cells were directly exposed to the living bacteria, while our approach focused on secreted bioactive molecules since only CMs were tested. Interestingly, the kinetics of ADRP and ANGPTL4 expression in the presence of *A. parvulum* and *P. copri* differs with a more persistent effect at 12 h for the later (Fig. 8). At the mRNA level, it appears that the two bacteria regulate gene expression at least at the same level as the agonist troglitazone.

Since phosphorylation plays an important role in the activation of nuclear receptors^35^,^36^ and has previously been demonstrated as a possible mechanism for PPARγ activation by bacteria^31^, we investigated the phosphorylation status of PPARγ. Our experiments showed that *A. parvulum* and *P. copri* can affect the phosphorylation status of endogenous PPARγ long enough to trigger an activation of its downstream target genes. Interestingly this phosphorylation was observed for both isoforms of PPARγ of which PPARγ2 is not phosphorylated in its non-activated state. The N-terminal site of both isoforms PPARγ1 and γ2 contains a MAPK site^37^ which was shown to exert either positive^38^ or negative^37^ effects on its transcriptional activity. This discrepancy in the role of PPARγ phosphorylation might be explained by the use of different cell models in the mentioned studies.

Three MAP-kinase pathways are at the crossroads of many cellular pathways. Members of the extracellular signal-regulated kinases (ERK1-2), are activated predominantly by growth factors^39^. In contrast, activity of Jun NH_2_-terminal kinase (JNK, also known as SAPK) and p38 kinase is increased by exposure of cells to environmental stress^40^. Since the MAPK have been reported to phosphorylate PPARγ for downstream signaling^37^ we investigated the involvement of these important kinases, involved among others in inflammation.

We identified the involvement of the ERK1/2 pathway using the specific inhibitor U0126 on the PPARγ reporter cell line. Moreover, we confirmed that observation at the protein level in the parental HT-29 cell line. Our results indicate the involvement of ERK1/2 phosphorylation in the *A. parvulum* and *P. copri-*mediated activation of PPARγ in the intestinal epithelial cell line HT-29. The observed mechanism could link *A. parvulum* and *P. copri* to their host’s metabolism through the activation of the PPARγ target gene ANGPTL4, an important regulator of systemic lipid metabolism. Interestingly, ANGPTL4 regulation by commensals^31^ and probiotics^41^ has been described, and we recently showed that SCFA were among its key modulators^25^. Our study reveals the presence of additional compounds with the potential to regulate PPARγ activity and its target gene expression in the intestine.

A first assessment on the nature of the active compounds underlines again the diversity of bacterial compounds capable to modify human transcriptional regulation. Although both compounds were inactivated by heat treatment (Fig. 9), suggesting molecules susceptible to denaturation, hydrolysis or evaporation, they clearly differ in size. While *P. copri* produces a small PPARγ activating molecule with a size between 1 and 3 kDa, corresponding to the size of a membrane lipid, *A parvulum* activates PPARγ through a molecule larger than 100 kDa which could be a secreted protein or large fragments of cell wall materials (Fig. 10).

PPARγ has been attributed protective roles against inflammation and even cancer in the GI tract^16^,^42^,^43^,^44^,^45^. The link between inflammatory bowel diseases and PPARγ as well is now well established^46^. Thus, the identification of bacterial strains able to regulate PPARγ activity in the gut is of significant patho-physiological and therapeutic interest. We believe that bacterial compounds may be the most relevant bioactive products in this context. The presented study describes novel mechanisms through which conditioned media from specific human gut bacteria can regulate PPARγ in intestinal epithelial cell lines *in vitro* and emphasizes the functional differences in activation mechanisms. In the presented case of the studied of *P. copri* and *A. parvulum* it is difficult to extrapolate the role of the found effect in the intestinal ecosystem as a whole. The entanglement of this mechanism not only in complex physiological conditions but also in the context of intestinal inflammation remains to be studied rigorously. However, the importance of this finding is underlined by the fact that both strains have been linked to inflammatory conditions when overrepresented. Increased presence of *P. copri* in the intestine has been linked to arthritis^47^ and *A. parvulum* linked to periodontitis^48^. Reports of local enrichment of specific species in sub-niches of the intestinal tract support the importance of single bacteria in host-microbiota interactions^49^.

The presented findings could contribute to the conception of new tailored-made approaches to ameliorate human health through directed interventions in the intestinal microbiota. Further studies have and will continue to document the systemic influences of the gut microbiota eventually leading to an integrative understanding of the intestinal microbiota and its role in human health.

## Materials and Methods

### Cell Culture and Reagents

The human epithelial cell lines HT-29 were obtained from the American Type Culture Collection (ATCC, Rockville, MD), HT-29 cells were grown in RPMI 1640 supplemented with 2 mM L-glutamine, 50 IU/mL penicillin, 50 μg/mL streptomycin and 10% heat-inactivated fetal calf serum (FCS) in a humidified 5% CO_2_ atmosphere at 37 °C. All culture media were supplied by Lonza. All agonists and inhibitors were dissolved in DMSO following the manufacturer’s recommendations. PPARγ activator TDZs: pioglitazone (Pio, 5 μM), rosiglitazone (Rosi, 10 μM), troglitazone (Tro, 5 μM) were from Cayman Chemicals and were used interchangeably due to their same level of activation on the used reporter system. MAPK kinase inhibitor: U0126 (MEK1/2) was purchased from Calbiochem. Butyrate was used at 2 mM except in the dose-response experiment where a range of concentrations from 0.5 to 8 mM was assessed for all the compounds tested (acetate, butyrate, propionate, formate, lactate and succinate (Sigma).

### Culture of Commensal Strains and Preparation of Conditioned Media

57 commensal strains were selected from the in-house INRA strain collection of human intestinal bacteria and grown in anaerobic conditions at 37 °C using the Hungate culture method^50^. Screened strains and corresponding growth media are listed in Lakhdari *et al*.^23^. Bacterial cultures were centrifuged at 5,000 × g for 10 min. Conditioned media (CM) were then collected and filtered on 0.2 μm PES filters. Non-inoculated bacteria culture medium served as control. Concentrations of organic acids produced were assessed by HPLC and gas chromatography as described^23^.

### Heat treatment and fractioning of Conditioned Media

To characterize the nature of the active compound conditioned media were exposed twice to high temperature (100 °C for 10 min) and subsequently cooled down on ice. Native conditioned media was size-fractionated using 100 kDa, 50 kDa, 30 kDa, 10 kDa, 3 kDa and 1 kDa cut-off filters (Millipore) whereby the flow-through was harvested and applied to the next smaller filter. All preparations were used for PPARγ activation testing as described below.

### Plasmid Construction and Production of Stable PPAR-luciferase Reporter Cell-Lines

Previously published PPARγ reporter construct pJ3-TK-Luc (kind gift from M. Chamaillard, INSERM Lille, France) was used to establishing HT-29-PPARγ reporter cell-lines. pTK-Hygro (Invivogen) was co-transfected with pJ3-TK-Luc using TFX50™ (Promega), according to manufacturer’s recommendations. Stable reporter cell lines for PPARγ were selected using Hygromycin (600 μg/ml, InvivoGen) and validated using rosiglitazone.

### Analyses of PPARγ activation: Luciferase Reporter Assay

For each experiment, HT-29-PPARγ reporter cells^51^ were seeded at 2.5 × 10^4^ cells per well in 96-well plates. After 24 h of culture, cells were stimulated for 24 hours with 10 μl of CM in a total culture-volume of 100 μl per well (i.e., 10% vol/vol). The screening was performed twice in triplicates. Follow-up experiments were performed in triplicates and repeated at least three times. Luciferase activity was quantified as relative luminescence units using a microplate reader (Infinite 200, Tecan) and the ONE-Glo™ Luciferase Assay System (Promega) according to the manufacturer’s instructions.

### Real-Time PCR

Cell lines were seeded in 24 well culture plates at densities of 0.5 × 10^6^ cells per well and cultured for 24 h before stimulation. After a stimulation time of 6 and 12 h, total RNA was extracted using RNeasy mini-Kit (Qiagen). cDNA was synthesized from 1 μg of RNA using the High-Capacity cDNA Archive Kit (Applied Biosystems). qPCRs were carried out using an ABI Prism 7700 (Applied Biosystems) thermal cycler in a reaction volume of 25 μl. mRNA was quantified using SYBR Green (Applied Biosystems)-based quantitative real-time PCR for adipose differentiation-related protein (ADRP), Angiopoietin-like protein 4 (ANGPTL4). β-Actin was used for normalization. Samples were tested in experimental duplicates and biological triplicates. Data are presented as fold change in the relative gene expression. Primer sequences: ADRP; F-CTGTTCACCTGATTGAATTTGC, R-AGAGCTTATCCTGAGCATCCTG, ANGPTL4; F-AAAGAGGCTGCCCGAGAT, R-TCTCCCCAACCTGGAACA, β-actin; F-CCTGGCACCCAGCACAAT, R-GCCGATCCACACGGAGTACT.

### Western blot analysis

HT-29 cells were seeded at densities of 5 × 10^5^ cells per well in 24-well-plates and starved for 24 h before stimulation. Cells were treated according to figure descriptions and subsequently nuclear and cytoplasmic extracts were prepared with NE-PER Nuclear and Cytoplasmic Extraction Reagent Kit (Pierce) according to the manufacture instructions. To prevent dephosphorylation and protein degradation in prepared extracts 1 x Complete Protease Inhibitor Cocktail and PhosSTOP (Roche) were used. Proteins were quantified by Bradford (BioRad). Samples were resolved in a denaturing 10% polyacrylamide gel and transferred to a nitrocellulose membrane (Hybond ECL, GE Healthcare). Western blots were probed with anti-phospho-PPARγ (Ser-82) (Milipore, Rabbit mAb clone AW504), total PPARγ (Santa Cruz, mice mAb, clone E-8), ERK1/2 (Cell signaling, rabbit mAb clone 137F5) followed by polyclonal rabbit or mouse horseradish peroxidase-coupled antibody (DAKO). A monoclonal anti-GAPDH-peroxidase (Sigma) was used as loading control. Finally, protein bands were revealed using the ECL™ detection system (Amersham Pharmacia Biotech) according to the manufacturer’s instruction.

### Statistical Analysis

Presented results are representative of at least 3 independent experiments. Results are expressed as mean ± SEM of representative triplicate measurement. Data were analyzed using Student’s t-test or ANOVA testing followed by post-hoc Tukey testing Principal component analysis (PCA) and clustering analysis were performed using ade4 and pvclust R packages respectively. Monte Carlo permutation test was performed (1000 repetitions) to assess the significance of the clustering performed. Correlations were calculated using two-sided Spearman testing.

## Additional Information

**How to cite this article:** Nepelska, M. *et al*. Commensal gut bacteria modulate phosphorylation-dependent PPARγ transcriptional activity in human intestinal epithelial cells. *Sci. Rep.* **7**, 43199; doi: 10.1038/srep43199 (2017).

**Publisher's note:** Springer Nature remains neutral with regard to jurisdictional claims in published maps and institutional affiliations.

## Supplementary Material

Supplementary Table and Figures
