# Supplementary material for: Commensal gut bacteria modulate phosphorylation-dependent PPARγ transcriptional activity in human intestinal epithelial cells
Source: Sci Rep. 2017 Mar 7;7:43199. doi: 10.1038/srep43199 (PMC5339702; doi:10.1038/srep43199)

Supplementary material.

Commensal gut bacteria modulate phosphorylation-dependent PPARγ transcriptional activity in human intestinal epithelial cells

Malgorzata Nepelska,[a] Tomas de Wouters,[a,b] Elsa Jacouton,[a] Fabienne Béguet-Crespel,[a] Nicolas Lapaque,[a] Joël Doré,[a,c], Velmurugesan Arulampalam,[d], Hervé M. Blottière, [a,c*]

SUPPLEMENTARY TABLE 1 : List of all tested commensal bacterial strains and OD600 (growth), pH of conditioned medium, organic acid concentration and PPAR gamma activity (as fold increase as compared to control medium).

					organic acid concentrations [mM]							
species	code	phylum	OD600	ph	pyruvate	lactate	formate	acetate	propionate	butyrate	PPARgamma activity	SD
P. acnes	B15	Actinobacteria	0,84	4,82	0	51,795	0,95	0	0	0	1,18	0,16
E. rectale	ATCC 33656 - (	Firmicutes	0,2309	6,6	0,87	14,9	2,345	0	13,98	0	1,84	0,09
R. hansenii	DSM 20583	Firmicutes	0,451	4,83	0,195	36,49	0	0	0	0	0,99	0,09
S. ruminantium	ATCC19205 - D	Firmicutes	0,526	6,36	7,53	4,39	0	0	0	0	0,93	0,31
R. faecis	DSM 16840	Firmicutes	0,6233	5,46	0	19,715	2,95	0	5,655	7,935	3,80	0,16
R. hominis	DSM 16839	Firmicutes	0,68	4,62	1,44	30,63	0	0	0	8,92	5,11	1,43
R. intestinalis	DSM 14610	Firmicutes	0,218	4,98	0,205	18,775	0	0	0	11,72	5,40	1,33
R. obeum	ATCC 29174	Firmicutes	0,472	6,52	7,37	1,45	0	0,985	0	0	1,00	0,04
R. lactaris	ATCC 29176	Firmicutes	0,565	6,48	7,545	1,34	0	1,03	0	0	0,96	0,11
R. gnavus	FRE1 ou souch	Firmicutes	0,517	6,24	5,95	1,99	0	1,68	0	0	0,97	0,04
A. parvulum	DSM 20649T	Actinobacteria	0,653	4,32	0,2	70,07	0,55	2,555	0	0	1,27	0,06
B.breve I	DSMZ 20091	Actinobacteria	0,52	5,54	2,125	20,22	0,745	2,72	8,07	0	0,85	0,48
C. sardiniensis	C3PR	Firmicutes	0,313	7,36	0,57	37,205	0	3,02	0	7,8	2,56	0,20
F. prauznitii	L2-6	Firmicutes	0,511	6,48	0	15,77	5,2	3,295	5,965	5,275	2,54	0,17
C. sardiniensis	I11PR	Firmicutes	0,343	7,45	0,07	27,585	0	3,88	0	7,97	2,56	0,53
E. formicigene	ATCC 27755	Firmicutes	0,56	6,2	7,11	1	0	4,875	0	3,61	1,20	0,88
C. aerofaciens	ATCC 25986	Actinobacteria	0,1906	6,53	0,58	16,355	4,105	5,67	0	0	1,47	0,06
C. sordellii	ATCC 9714	Firmicutes	0,144	6,71	0,4	13,355	2,795	5,67	0	0	1,09	0,33
C. sardiniensis	C9PR	Firmicutes	0,288	7,32	0,37	37,165	0	6,145	0	3,75	2,25	0,09
C. sardiniensis	D12PR	Firmicutes	0,398	7,45	0,455	24,69	0	6,63	0	5,98	2,93	0,36
C. sardiniensis	C7PR	Firmicutes	0,387	7,41	0,46	17,78	0	6,89	0	8,335	3,45	0,16
B. fragilis	B6 - AL 2553	Bacterioides	0,4961	6,07	0	13,515	3,31	6,945	16,005	0	1,93	0,27
B. fibrisolvens	DSM3071	Firmicutes	0,6596	5,21	0	44,2	3,72	7,135	4,455	0	1,27	0,23
R. torques	ATCC 27756	Firmicutes	0,318	5,49	0,505	16,34	2,17	7,195	0	0	1,24	0,06
C.paraputrificu	G12PR - X7344	Firmicutes	0,1961	6,18	0,195	11,275	5,33	7,615	0	2,84	2,61	0,32
F. naviforme	DSM 20699	Fusobacterium	0,644	6,92	0,12	11,43	1,31	7,96	1,81	23,9	5,33	0,36
B. caecae	ATCC 43185 - (	Bacterioides	0,4864	5,77	0	12,025	2,92	8,72	14,09	0	2,31	0,29
C. leptum	ATCC29065 - D	Firmicutes	0,2205	5,92	0	26,285	5,36	8,945	4,835	0	1,01	0,14
B. uniformis	ATCC 8492	Bacterioides	1,094	4,82	1,855	14,15	0,81	8,995	43,485	0	2,58	1,03
B. thetaiotaom	ATCC 29148 - V	Bacterioides	0,766	5,26	0,39	7,03	0,615	9,55	0	0	1,01	0,07
P. johnsonii	DSM 18315	Bacterioides	0,598	5,14	0,17	6,41	0	9,565	0	0	1,09	0,23
B. vulgatus	ATCC 8482 - Cl	Bacterioides	0,4356	5,91	0	11,785	3,5	9,9	15,295	0	1,67	0,34
B. ovatus	ATCC 8483 - Cl	Bacterioides	0,678	5,08	0,265	9,35	0,7	10,115	0	0	1,20	0,10
P. distasonis	CIP104284 - AT	Bacterioides	0,665	6,19	0,135	3,67	6,13	10,23	41,775	0	4,21	0,10
C. nexile	ATCC 27757 - D	Firmicutes	0,2979	6,63	0,17	9,675	4,66	10,45	6,23	0	0,95	0,17
B. dorei I	D8	Bacterioides	0,737	4,96	0,155	10,435	0,77	10,68	0	0	1,13	0,11
C. sardiniensis	D7PR	Firmicutes	0,5694	6,66	0	1,59	3,135	12,18	4,22	10,44	4,35	0,42
B. doreill	DSM 17855	Bacterioides	0,987	4,95	0,14	7,61	0	12,63	0	0	1,07	0,20
B. breve II	ATCC 15701 - 8	Actinobacteria	0,313	5,14	0,67	8,725	2,82	13,155	0	0	1,55	0,11
F. nucleatum	ATCC 51190 - D	Fusobacterium	0,398	6,81	0,12	36,955	0	16,73	0	14,245	2,38	0,97
P. copri	DSM 18205	Bacterioides	0,444	4,91	1,675	15,565	3,2	17,915	0	0	1,44	0,09
R. productus	DSMZ 2950 - 9	Firmicutes	0,552	4,97	0,185	7,935	0	21,075	0	0	1,29	0,10
C. sporosphaer	ATCC 25781	Firmicutes	0,463	6,94	0,455	12,36	0	21,725	17,365	1,7	2,41	0,40
C. coccoides	ATCC 2936	Firmicutes	0,4498	5,11	0	15,695	2,275	25,17	4,61	0	1,01	0,06
B. dentium II	ATCC 27534 - 8	Actinobacteria	0,797	4,37	0,475	27,39	4,805	37,91	0	0	1,78	0,18
B. gallicum	ATCC 49850 - (	Actinobacteria	0,583	4,12	7,95	10,85	2,495	40,605	0	0	0,65	0,64
B. dentium I	ATCC 15423	Actinobacteria	0,876	4,25	0,26	52,005	0,875	40,755	0	0	0,79	0,60
B. infantis	DSM20088/AT(	Actinobacteria	0,761	4,05	0,48	37	2,075	44,405	0	0	0,97	0,97
B. animalis	DSM 20104	Actinobacteria	0,685	4,09	0,95	36,535	1,975	46,25	0	0	0,84	0,83
B. longum	ATCC 15707 - N	Actinobacteria	0,896	3,92	0,315	54,62	0,59	53,06	0	0	0,16	0,16
B. catenulatum	ATCC27539 - C	Actinobacteria	1,066	3,9	0,275	61,62	0	53,15	0	0	0,13	0,13
B. ruminantium	ATCC 49390	Actinobacteria	1,075	3,87	0,28	60,87	0,375	54,565	0	0	0,10	0,10
B. bifidum	DSM20082/JCN	Actinobacteria	0,793	3,89	0,225	63,455	0,865	58,305	0	0	0,05	0,06
B. choerinum	DSM 20434	Actinobacteria	0,971	3,87	0,28	60,145	0,435	60,845	0	0	0,13	0,10
B. adolescentis	ATCC 15703	Actinobacteria	0,884	3,85	0,24	58,185	1,805	61,495	0	0	0,02	0,03
B. angulatum	ATCC 27535 - (	Actinobacteria	1,07	3,95	0,47	62,52	0,79	61,615	0	0	0,07	0,08
B. pseudocater	DSM 20438 - A	Actinobacteria	1,27	3,85	0,535	61,21	1,175	63,965	0	0	0,01	0,00

SUPPLEMENTARY FIGURE 1: Comparison of PPARγ activation capacity of the different bacterial phyla tested reveals highest activation potential in Fusobacteria and Firmicutes phylum. ANOVA and post hoc Tukey testing confirmed significant differences between the Actinobacteria and Firmicutes as well as Fusobacteria. Significance levels are indicated as follows: ***$P<0.001$, **$P<0.005$.

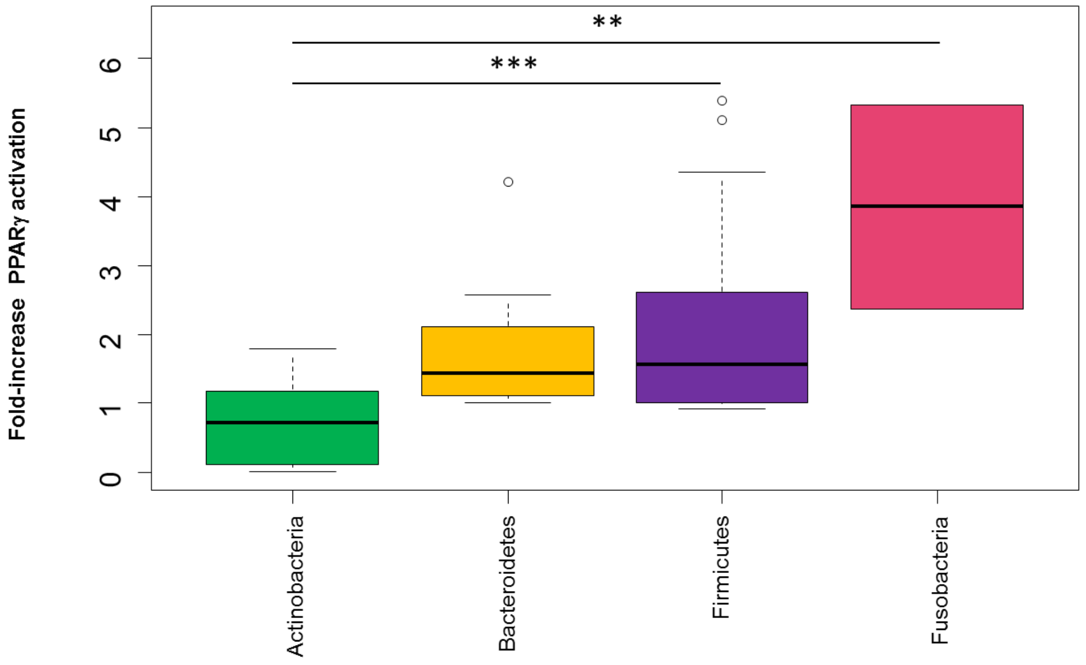

SUPPLEMENTARY FIGURE 2: Spearman correlation between different metabolites and PPARγ activation reveals correlations between different metabolites but only weak correlations between PPARγ activity and single metabolites except for butyrate (0.69) and propionate (0.41).

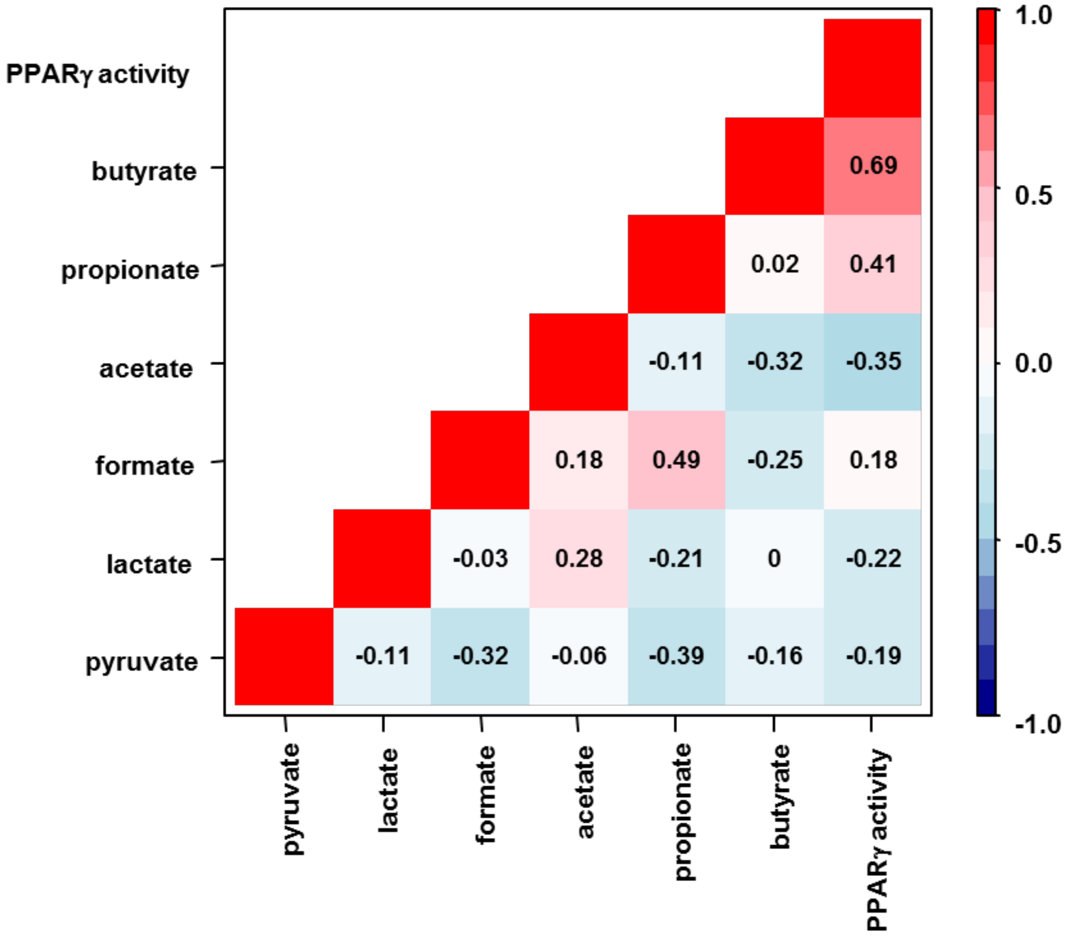

SUPPLEMENTARY FIGURE 3: Clustering using all metabolites as parameters reveals separation into one inhibitory group (cluster 1), a strongly activating group (cluster 2), a group with broad activation spectrum (cluster 3) and a mostly neutral group (cluster 4). Significance was tested using ANOVA and post hoc Tukey testing and is indicated using letters. Boxes with no shared letter differ significantly with $P < 0.05$

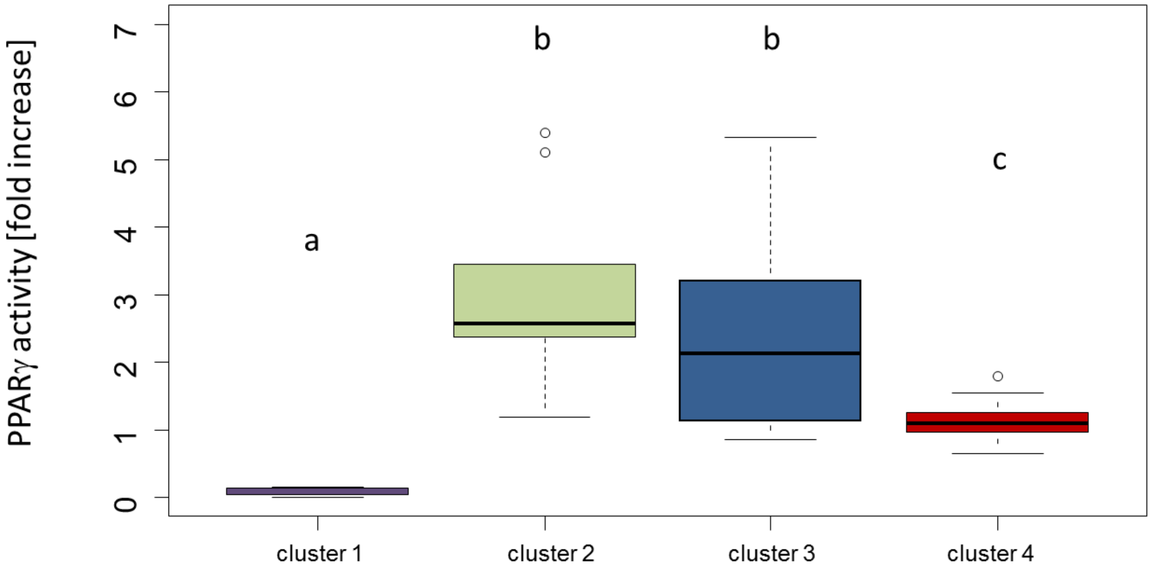

Supplement: Supplementary Table and Figures [file srep43199-s1.pdf]
